# Evaluating the Global Impact of Stroke Awareness Month: A Serial Cross-Sectional Analysis

**DOI:** 10.7759/cureus.28997

**Published:** 2022-09-10

**Authors:** Kashish Goyal, Aniket Nafri, Mahima Marwah, Saikumar Aramadaka, Pranshul Aggarwal, Sakshi Malhotra, Raam Mannam, Oman Gupta, Kashish Malhotra

**Affiliations:** 1 Department of Internal Medicine, Dayanand Medical College and Hospital, Ludhiana, IND; 2 Department of Internal Medicine, Adesh Institute of Medical Sciences and Research, Bathinda, IND; 3 Department of Internal Medicine, Narayana Medical College, Nellore, IND; 4 Department of Obstetrics and Gynaecology, Pandit Bhagwat Dayal Sharma Post Graduate Institute of Medical Sciences, Rohtak, IND

**Keywords:** healthcare awareness event, public health, global disparities, twitter, digital impact, stroke awareness month, stroke

## Abstract

Introduction: Stroke is the second-leading cause of mortality in the world and ranks fifth in terms of causes of death in the United States. “Time is brain” when it comes to the detection and treatment of a stroke as it can reduce morbidity and disability in the long run. May is recognized as Stroke Awareness Month to involve the concerned stakeholders. The goal of this month is to raise public awareness of the risk factors for stroke and to minimize its occurrence. We, for the first time, evaluated the actual impact of this awareness campaign to formulate evidence-based recommendations to promote stroke awareness.

Methods: The total number of tweets posted in the month of May from 2014 to 2022 were extracted. The search queries used were “stroke awareness month OR stroke month OR #strokemonth OR #strokewarenessmonth” and “stroke OR #stroke”. Social network analysis of the tweets was done to understand the context of posts. Network analysis provides the capacity to estimate complex patterns of relationships and gives insights into useful information about impact, reach, and interactions in an environment. The top 100 related hashtags, influencers, and keywords were extracted. Beyond social media usage, Google Trends web search analysis was done for the search term ‘stroke awareness month’ for interest by region of the last five years to get an overall idea of the internet search trends globally.

Results: Out of the total 989,935 tweets about stroke posted in May 2022, only 1.07% of the tweets were specific to Stroke Awareness Month. The mean and standard deviation of the percentage of targeted action from 2014 to 2022 have been 3.14% and 1.35%, respectively. Forty-five percent of the top users never collaborated with each other. On Google Trends analysis, the event had primary involvement from the United States and the United Kingdom. The event had very limited reach in other continents, especially in Asian and African countries.

Conclusion: Our estimates highlight the limited digital impact of Stroke Awareness Month globally. The use of social media should be promoted, particularly in developing countries, to provide reliable information and generate user involvement on a global scale. Findings from this study can be leveraged to inform future policies for stroke awareness campaigns that improve public and global health.

## Introduction

A stroke, also known as a cerebrovascular accident, refers to the sudden development of a neurologic deficit that can be ascribed to a specific vascular cause lasting for more than 24 hours. It is the second-leading cause of mortality in the world, accounting for over 12 million incident cases of stroke in 2019, showing an increase of 70% since 1990 [1,2]. Similarly, in the United States, stroke ranks fifth in terms of causes of death with over 700,000 people developing stroke annually [2,3]. 

Early detection and treatment of a stroke are crucial because they can reduce morbidity and disability in the long run; as popularly stated, “time is brain” [4]. The person who calls for help is frequently a family member or a bystander because acute stroke patients may develop anosognosia or have a lack of knowledge. Therefore, everyone needs to be aware of and learn the red flags of stroke, which may be done by simply learning the acronym FAST (facial drooping, arm weakness, speech difficulties, and time to call emergency services) [5]. There is a significant lack of public awareness of the risk factors and warning symptoms of stroke, as demonstrated in several studies [6,7].

Hence, to involve the concerned stakeholders, May is recognized as Stroke Awareness Month. Following the signing of Presidential Proclamation 5975 by President George H. W. Bush in May 1989, Stroke Awareness Month was established in the United States [8]. The goal of this month is to raise public awareness of the risk factors for stroke and to minimize its occurrence. We, for the first time, evaluated the actual digital impact of this awareness campaign to formulate evidence-based recommendations to promote stroke awareness.

## Materials and methods

The total number of tweets posted in the month of May from 2014 to 2022 were extracted using the Twitter application programming interface (API) via Sprout Social [9]. The search queries used to retrieve tweets were “stroke awareness month OR stroke month OR #strokemonth OR #strokewarenessmonth” and “stroke OR #stroke”. The first search query was specific to Stroke Awareness Month and the second search query was a broad non-specific query to get an overall estimate of the total stroke-related tweets. No geographical or language restrictions were set.

Social network analysis of the tweets was done using Socioviz [10] to understand the context of tweets posted for the search query “Stroke or #Stroke” on the last day of Stroke Awareness Month. Network analysis provides the capacity to estimate complex patterns of relationships and gives insights into useful information about impact, reach, and interactions in an environment [11,12]. The top 100 related popular hashtags, influencers, and keywords were extracted.

In the network analysis, each entity was represented with a circle (a node) and was connected to other entities when there were interactions between them. The node size was set proportional to the number of retweets and mentions received, indicating the influence of a particular person in a network of conversations. Different colours represented different clusters of arguments or communities that frequently go together. ForceAtlas2’s model was used to show clusters which simulate a physical system to spatialize a network. Similarly to charged particles, nodes repel each other, while edges attract each other, like springs. The final configuration helps interpret the data since it produces a movement that converges to a balanced state [13].

Beyond social media usage, Google Trends web search analysis was done for the search term “Stroke Awareness Month” for interest by region over the last five years to get an overall idea of the internet search trends globally [14]. The worldwide demographic trends were studied using a normalized measure of the search term by relative search popularity in the set time range.

## Results

**Figure 1:**
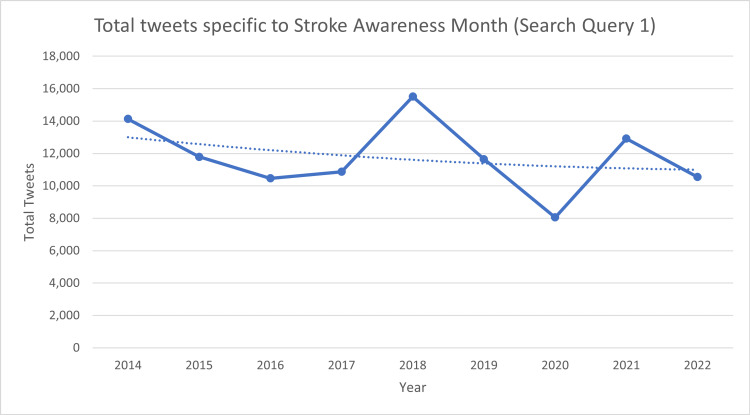
Total tweets specific to Stroke Awareness Month posted in May from 2014 to 2022. Search Query 1 was “stroke awareness month OR stroke month OR #strokemonth OR #strokewarenessmonth”.

**Figure 2:**
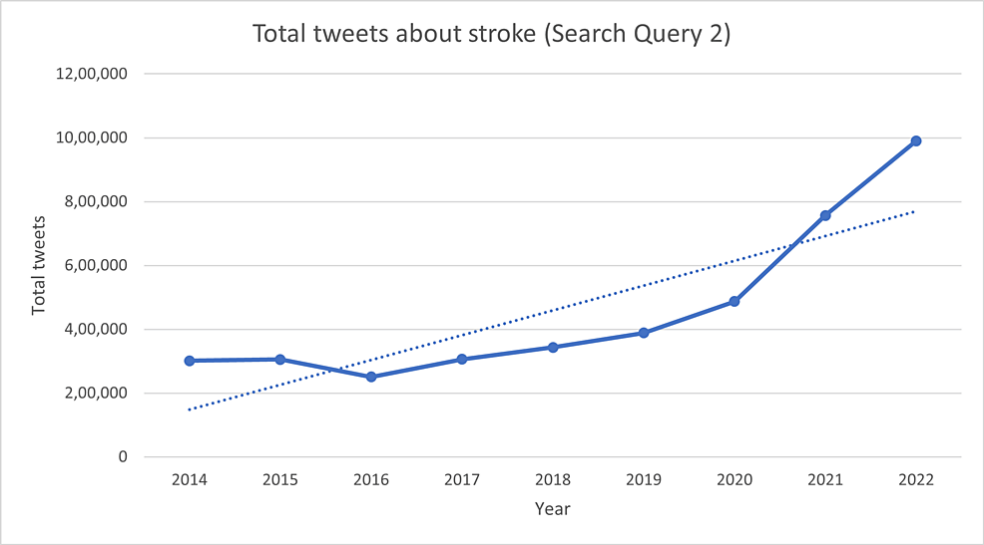
Total tweets related to stroke posted in May from 2014 to 2022. Search Query 2 was “stroke OR #stroke”.

Out of the total 989,935 tweets about stroke posted in May 2022, only 1.07% of the tweets were specific to Stroke Awareness Month. Though the total number of stroke-related tweets has been increasing year on year, the same cannot be said for Stroke Awareness Month-related tweets. The mean and standard deviation of the percentage of targeted action from 2014 to 2022 have been 3.14% and 1.35%, respectively. A spike of total tweets targeting Stroke Awareness Month has been seen in the first few days at the start of May which corresponds to the start of Stroke Awareness Month. Table 1 shows the total tweets related to stroke and Stroke Awareness Month. Figure 1 shows the trends of tweets specific to Stroke Awareness Month from 2014 to 2022 for the search query “stroke awareness month OR stroke month OR #strokemonth OR #strokewarenessmonth”. Figure 2 shows the trends of tweets specific to stroke from 2014 to 2022 for the search query “stroke OR #stroke”.

**Table 1:** Total tweets posted in the month of May from 2014 to 2022 with search queries related to Stroke Awareness Month and stroke. The "search query 1" was specific to Stroke Awareness Month. The "search query 2" was a broad non-specific query to get an overall estimate about the total stroke related tweets.

Year	Search Query 1: "stroke awareness month OR stroke month OR #strokemonth OR #strokewarenessmonth"	Search Query 2: "stroke OR #stroke"	Percentage (Search Query 1/ Search Query 2)
2014	14,130	3,01,056	4.69%
2015	11,789	3,04,998	3.87%
2016	10,463	2,50,815	4.17%
2017	10,872	3,05,843	3.55%
2018	15,517	3,43,116	4.52%
2019	11,655	3,88,436	3.00%
2020	8,058	4,86,788	1.66%
2021	12,919	7,56,306	1.71%
2022	10,551	9,89,935	1.07%

**Figure 3:**
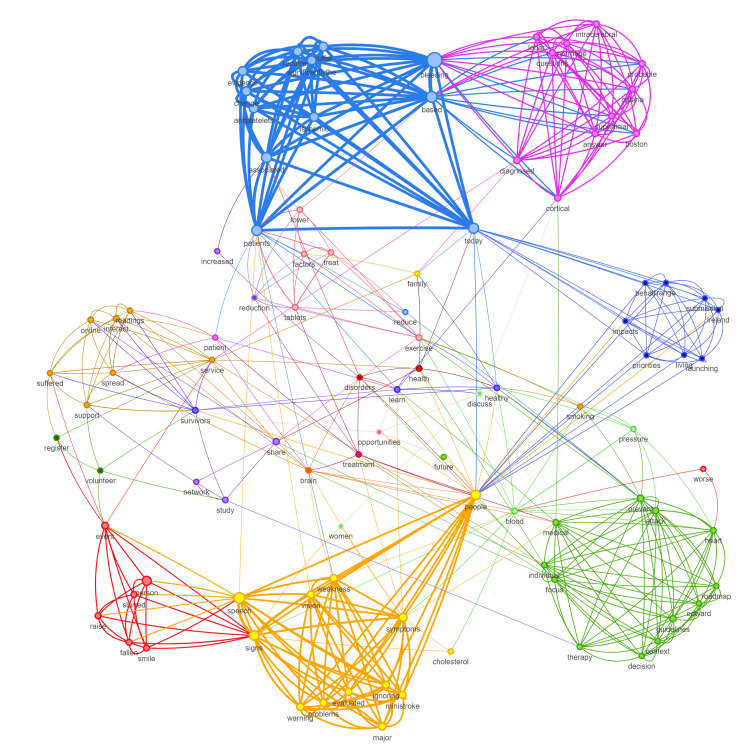
Social network analysis of the top 100 most commonly associated keywords with the search query "stroke OR #stroke".

On social network analysis, it was seen that 45% of the top users never collaborated with each other. The top five hashtags associated with our search query were ‘#doac’, ‘#harvardhealth’, ‘neurotwitter’, ‘neurology’, and ‘brainhealth’. The social network analysis of the top associated keywords is shown in Figure 3. On Google Trends analysis, the event had primary involvement from the United States and the United Kingdom. The event had very limited reach in Asian and African countries.

## Discussion

Though in absolute terms, over ten thousand tweets were posted about Stroke Awareness Month in 2022, when comparing it relatively to the total tweets posted about stroke, it can be seen that the impact of Stroke Awareness Month is limited. With just under 5% of the total tweets aimed at creating awareness about a life-threatening condition like stroke, targeted actively driven effort by all the stakeholders is needed to prioritize and promote Stroke Awareness Month globally.

Furthermore, tweeting about stroke awareness was largely a singular event and not consistent throughout the month which was also seen in a similar study analyzing the social media trends of Breast Cancer Awareness Month [15]. The lack of targeted action to promote Stroke Awareness Month is consistent with a similar article that compared the digital activity of Deep Vein Thrombosis Awareness Month [16]. With limited literature on the actual real-life impact of healthcare awareness events [17,18], the associations of top-linked keywords and hashtags may help in identifying the recurring themes and unmet needs in the perception of stroke awareness and stroke management globally.

As Stroke Awareness Month currently has a limited impact beyond the United States and the United Kingdom, active partnerships are needed to amalgamate communities and ensure collaboration amongst all the concerned stakeholders. Media outlets also play a vital role in directing the positive narrative and starting conversations globally. The utility of dedicated themes each year for global campaigning about stroke awareness needs to be further evaluated. Constructive feedback from highly impactful events such as World Hypertension Day shall be taken to steer equitable policy development [19].

Though the limited digital participation of African countries was also seen in other similar articles evaluating the impact of Hernia Awareness Month [20] and Polycystic Ovary Awareness Month [21], improving representation from less privileged communities may help to bridge the gap between economic and social disparities in stroke awareness.

However, our results shall be considered an underestimation as the data of other social media platforms such as Facebook and Instagram were not retrieved because of a lack of access to API to extract historical data globally. Furthermore, the data of private profiles were not available. Further studies shall focus on understanding the cultural and programmatic differences in the campaigning of Stroke Awareness Month in various countries. It shall be noted that the Indian Medical Organization (IMA) recognizes October as Stroke Awareness Month [22]. It needs to be further studied whether campaigning of stroke awareness in different months in various countries helps in an overall increase in the impact of this awareness event at a global level.

## Conclusions

Our estimates highlight the limited digital impact of Stroke Awareness Month globally. It is crucial for everyone to understand stroke, identify it, and take precautions to reduce risks for themselves and their families because a stroke can have a life-altering effect with significant disability. To have a major impact, it is crucial that there be widespread action and campaigning to support this healthcare awareness month. Social media should be used as a medium for spreading reliable information and generating user involvement on a global scale. The positive input from these findings shall be used to build future policies for other awareness campaigns that improve public and global health.
